# *Agrobacterium rhizogenes*-mediated transformation of *Superroot*-derived *Lotus corniculatus *plants: a valuable tool for functional genomics

**DOI:** 10.1186/1471-2229-9-78

**Published:** 2009-06-25

**Authors:** Bo Jian, Wensheng Hou, Cunxiang Wu, Bin Liu, Wei Liu, Shikui Song, Yurong Bi, Tianfu Han

**Affiliations:** 1 The National Key Facility for Crop Gene Resources and Genetic Improvement (NFCRI), Institute of Crop Sciences, The Chinese Academy of Agricultural Sciences, Beijing 100081, PR China; 2 School of Life Sciences, Lanzhou University, Lanzhou, Gansu 730000, PR China; 3 Current address: Department of Biology, Norwegian University of Science and Technology, Realfagbygget, Trondheim NO-7491, Norway

## Abstract

**Background:**

Transgenic approaches provide a powerful tool for gene function investigations in plants. However, some legumes are still recalcitrant to current transformation technologies, limiting the extent to which functional genomic studies can be performed on. *Superroo*t of *Lotus corniculatus *is a continuous root cloning system allowing direct somatic embryogenesis and mass regeneration of plants. Recently, a technique to obtain transgenic *L. corniculatus *plants from *Superroot*-derived leaves through *A. tumefaciens-*mediated transformation was described. However, transformation efficiency was low and it took about six months from gene transfer to PCR identification.

**Results:**

In the present study, we developed an *A. rhizogenes*-mediated transformation of *Superroot*-derived *L. corniculatus *for gene function investigation, combining the efficient *A. rhizogenes*-mediated transformation and the rapid regeneration system of *Superroot*. The transformation system using *A. rhizogenes *K599 harbouring pGFPGUS*Plus *was improved by validating some parameters which may influence the transformation frequency. Using stem sections with one node as explants, a 2-day pre-culture of explants, infection with K599 at OD_600 _= 0.6, and co-cultivation on medium (pH 5.4) at 22°C for 2 days enhanced the transformation frequency significantly. As proof of concept, *Superroot*-derived *L. corniculatus *was transformed with a gene from wheat encoding an Na^+^/H^+ ^antiporter (*TaNHX2*) using the described system. Transgenic *Superroot *plants were obtained and had increased salt tolerance, as expected from the expression of *TaNHX2*.

**Conclusion:**

A rapid and efficient tool for gene function investigation in *L. corniculatus *was developed, combining the simplicity and high efficiency of the *Superroot *regeneration system and the availability of *A. rhizogenes*-mediated transformation. This system was improved by validating some parameters influencing the transformation frequency, which could reach 92% based on GUS detection. The combination of the highly efficient transformation and the regeneration system of *Superroot *provides a valuable tool for functional genomics studies in *L. corniculatus*.

## Background

Legume crops are economically important in supplying oil and protein for human consumption and animal forage, and are also major contributors to the global nitrogen cycle due to their unique ability of symbiotic nitrogen fixation. Besides their agricultural importance, legumes also produce a variety of beneficial secondary compounds, many of which have been proved to have health-promoting properties such as providing protection against human diseases [1,2].

Plant transformation is a useful tool in molecular analysis of gene function and limited transformation capability constitutes a significant barrier in making advances in our understanding of gene functions [3]. In legumes, *A. tumefaciens*-mediated transformation is the method of choice to test gene functions [4]. However, many cultivated grain legumes are still recalcitrant to current transformation technologies or show low transformation frequencies which limit their potential as objects for gene functional studies [5].

*A. rhizogenes*, a soil-borne bacterium, causes the production of hairy roots at the wounding sites. It transfers T-DNA from the Ri plasmid into the plant genome and also T-DNA of the binary vector when co-transferred [6,7], allowing the integration of a foreign gene. Hairy roots have the unique property of being able to grow *in vitro *in the absence of exogenous plant growth regulators [8]. These growth characteristics and the high transformation frequency of *A. rhizogenes *have made the production of 'composite plants' *in vitro *and *ex vitro *a tool to test gene functions for root biology [8-10]. However, it does not allow assessing gene function on the whole plant level because of the non-transformed shoot parts. Additionally, not all the hairy roots are co-transformed [11], which makes the analyses complicated.

*L. corniculatus *is a perennial, fine-stemmed, leafy legume that has become of increased importance in agriculture as pasture and hay crops in recent years. It has the potential to become a major crop replacing white clover and alfalfa in temperate forage-producing regions of the world, because of its high nutritive value and its tolerance to adverse environmental conditions. A unique *in vitro *culture system of long-lived *Superroot *was reported in the legume *L. corniculatus *[12]. This system allows continuous root cloning, direct somatic embryogenesis and mass regeneration of plants without addition of exogenous plant growth regulators [13,14]. However, direct transformation of *Superroot *was unsuccessful, thus limiting its use. Recently, transgenic *L. corniculatus *was obtained from *Superroot*-derived leaves through *A. tumefaciens-*mediated transformation. However, the transformation efficiency was low, as calli were observed at the cuts of merely 56 leaf segments among 919 segments 50 days after transfer, and the process from gene transfer to PCR identification took six months [14]. Thus, the frequency and efficiency in *A. tumefaciens*-mediated transformation of *Superroot*-derived leaves still stand as a barrier for its extensive use.

In the present study, we developed a highly efficient *A. rhizogenes*-mediated transformation of *Superroot*-derived *L. corniculatus*, exploiting the combination of highly efficient *A. rhizogenes*-mediated transformation [5,10] and the rapid and simple regeneration system of *Superroot *[12-14]. This system can be used to study gene functions on the whole plant level. The improved transformation was achieved by optimizing parameters that influence the transformation efficiency, such as explant type [15,16] and pH of the co-cultivation medium (CCM) [17]. In order to further validate this system for gene function analysis, *TaNHX2 *[18], a gene from wheat encoding an Na^+^/H^+ ^antiporter that plays an important role in plant salt tolerance [19,20], was introduced into the *Superroot *of *L. corniculatus *and the salt tolerance of regenerated plants was assessed.

## Results

### Transgenic *Superroot *plants obtained from hairy roots induced by *A. rhizogenes *with high efficiency

**Figure 1:**
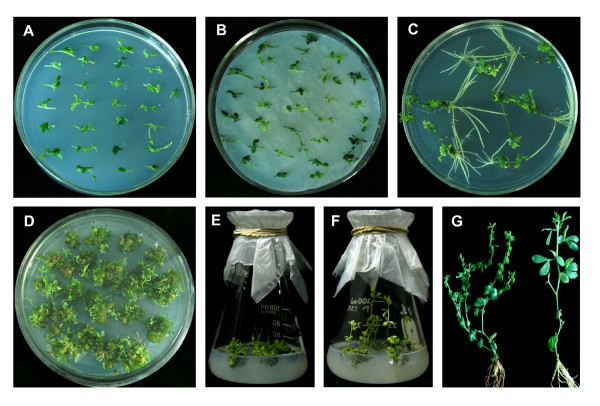
**Transgenic *L. corniculatus *cv. *Superroot *plants obtained from hairy roots induced by *A. rhizogenes***. Obtainment of transgenic *L. corniculatus *cv. *Superroot *by *A. rhizogenes *mediated transformation. Pre-cultivation of explants on MS medium (A). Co-cultivation of explants after infection with *A. rhizogenes *(B). Hairy roots began to appear at the wounding sites of the explants about 7 days after being transferred onto 1/2 MS medium. Pictures were taken 14 days after the first appearance of hairy roots (C). Plantlets/shoots regenerated from hairy roots on the RM 4 weeks later (D). Shoots were transferred onto MS medium for elongation (E). Shoot elongation and root formation about 4 weeks after being transferred onto MS medium (F). Comparison between transgenic *L. corniculatus *by *A. rhizogenes*-mediated transformation (left) and wild type plant (right) (G).

After being pre-cultured in MS medium (Figure 1A), the explants were infected with *A. rhizogenes *and then placed on solid CCM (Figure 1B). The explants were placed on 1/2 MS medium to induce the hairy roots after co-cultivation. Seven days later, hairy roots began to appear at the wounding sites of the explants (Figure 1C). When the hairy root grew to a length of 3 to 4 cm, each individual hairy root was labelled with numbers and an approximately 1 cm long segment was cut axenically from each hairy root to be used for GFP and GUS detection. The hairy roots thus identified as GUS and GFP positive were then excised from the original explants and transferred to the regeneration medium (RM). Nearly 100% of the hairy roots regenerated into shoot buds or plantlets about 25 days later (Figure 1D). The shoot buds were transferred to MS medium without any plant growth regulators for stem elongation and rooting (Figure 1E and 1F). Transgenic *L. corniculatus *plants were obtained in about two and a half months and the regenerated plants had a typical hairy root phenotype with short internode and wrinkled leaves (Figure 1G).

### Molecular characterization of transgenic hairy roots and regenerated plants

**Figure 2:**
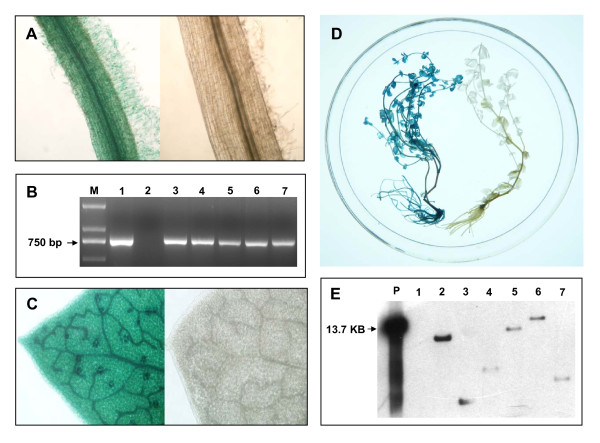
**GUS detection of hairy root and regenerated transgenic plants**. ×10 micrograph showing GUS staining of hairy root. Left panel, transgenic hairy root; right panel, negative control (A). PCR-amplification of *GUS *in regenerated plants (B). M, 1 kb DNA marker; 1, plasmid DNA; 2, negative control; 3–7, transgenic regenerated plants. ×20 micrograph showing GUS staining of leaf from a regenerated plant. Left panel, transgenic leaf; right panel, negative control (C). GUS staining of a regenerated transgenic plant (left) and a negative control (right) (D). Southern blot analysis of regenerated plants using a 750-bp GUS fragment as a probe (E). P, *Hin*d III-digested pGFPGUS*Plus *plasmid DNA; 1, negative control plant; 2–7, randomly selected transgenic regenerated plants. All negative controls were hairy roots or regenerated plants obtained through transformation mediated by *A. rhizogenes *harbouring no binary vector.

**Figure 3:**
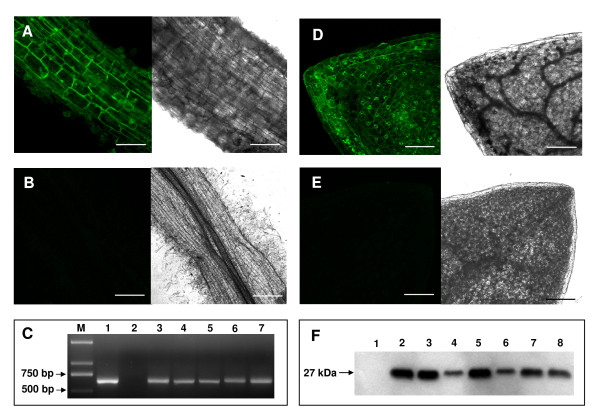
**GFP detection of hairy roots and regenerated transgenic plants**. GFP-derived fluorescence detected by laser scanning confocal microscopy in a transgenic hairy root, scale bar = 70 μm (A) and in a negative control hairy root, scale bar = 300 μm (B). PCR-amplification of *GFP *in regenerated plants (C). M, 1 kb DNA marker; 1, plasmid DNA; 2, negative control; 3–7, transgenic regenerated plants. GFP-derived fluorescence detected by laser scanning confocal microscopy in a leaf from a regenerated plant, scale bar = 150 μm (D) and in a leaf from a negative control plant, scale bar = 150 μm (E). Western blot assay for the detection of GFP protein levels in independent transgenic plants using an anti-GFP antibody (F). 1, negative control plant; 2–8, independent transgenic plants. All negative controls were hairy roots or regenerated plants obtained by *A. rhizogenes *harbouring no binary vector mediated transformation.

The hairy roots identified as being transgenic by GUS staining (Figure 2A) and GFP detection (Figure 3A) were transferred to RM for shoot induction. PCR analysis of the regenerated plants was performed with primers designed to amplify *GUS *and *GFP *fragments, respectively. The PCR results showed the presence of *GUS *(Figure 2B) and *GFP *(Figure 3C) bands of the expected sizes (750 and 641 bp, respectively) in the corresponding transgenic samples and their absence in the negative controls, indicating that all the positively transgenic hairy root-derived plants contained both the *GFP *and *GUS *genes.

Southern blot analysis was also carried out to identify the transgenic events. Genomic DNA of regenerated plants was digested with *Hind *III which cuts at a single site within the T-DNA. Restriction-digested DNA was then blotted and hybridized with a 750 bp digoxigenin (DIG)-labelled *GUS *fragment as a probe. As shown in Figure 2E, the six randomly selected regenerated plants showed a different single integration event of the T-DNA, thereby confirming their independent transgenic nature. No hybridization signal was observed in the control plant.

To further verify gene transfer, GFP and GUS expression were monitored on the whole plant level. In contrast to 'composite plants', in which only roots are transformed, the whole plantlets regenerated from the hairy roots showed GUS staining (Figure 2C and 2D) and GFP fluorescence (Figure 3D). The transformation events were additionally confirmed by Western blot using an anti-GFP antibody. As shown in Figure 3F, Western blot indicated the presence of GFP in randomly selected 7 independent transgenic plants with a band of about 27 kDa and no signal was detected in the control plant.

### Stem section with one node is the most suitable explant for transformation

**Figure 4:**
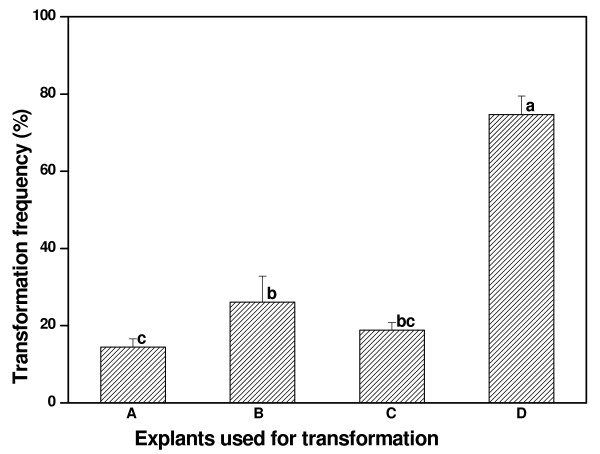
**Selection of the most suitable explant for *A. rhizogenes *mediated transformation of *L. corniculatus *cv. *Superroot***. Root (A), internode (B), leaf (C) and stem section with one node (D) were used as explants for transformation to find the most suitable explant. Means of transformation frequencies were compared using a Fisher's LSD test (P < 0.05) and column bars with the same letter are not significantly different. The experiment was performed in independent triplicate and each experiment contained about 30 samples.

Different types of explants may have diverse competence to *A. rhizogenes *infection. In the current study, root, leaf, internode and stem section with one node were used as explants to determine which type of explant is most suitable for *A. rhizogenes*-mediated transformation in *Superroot L. corniculatus*. The standard procedure described in Methods was used for this purpose with the preculture duration being one day, the nature of the explant being the only variable. As shown in Figure 4, the transformation frequency changed with explant types. The highest transformation frequency (74.64%) was obtained when the stem sections with one node were used as explants. In contrast, the transformation frequency was just 14.49% when roots were used as explants. The transformation frequency obtained with stem sections with one node as explants was significantly different (Fisher's Least Significant Difference (LSD) test; P < 0.05) to all other types of explants tested. Obviously, as the stem section with one node was the most suitable explant for *A. rhizogenes*-mediated transformation in *L. corniculatus *cv. *Superroot*, it was used to test the effects of other parameters on the transformation frequency.

### Effects of pre-culture duration on transformation frequency

Recent reports suggest that pre-culturing may influence the transformation frequency [15,17,21]. Prior to infection with *A. rhizogenes*, stem sections with one node were pre-cultured in MS medium for a varying period from 0 to 6 days, after which the standard procedure described in Methods was used for the remaining part of the assay.

**Figure 5:**
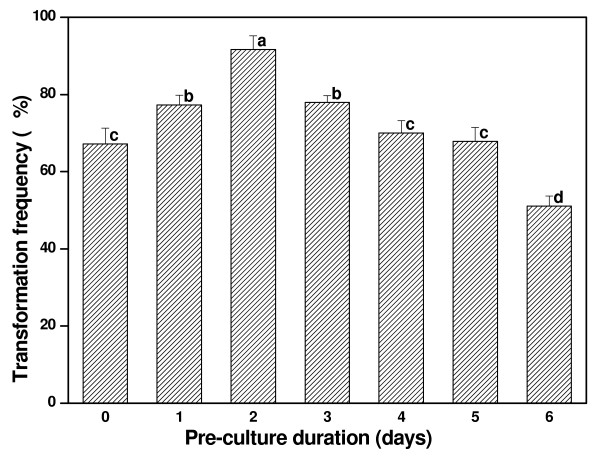
**Effect of pre-culture duration on transformation frequency**. A pre-culture duration ranging from 0 to 6 days was carried out to determine which one is most efficient. Means of transformation frequencies were compared using Fisher's LSD test and column bars with the same letter are not significantly different at P < 0.05. The experiment was performed in independent triplicate and each experiment contained about 30 samples.

Transformation frequency differed depending on pre-culture time as shown in Figure 5. The results demonstrated that the transformation frequency could be improved after 1 to 3 days pre-culture. The highest transformation frequency (91.67%) was observed after a 2 days pre-culture and it was remarkably different from the other pre-culture duration (P < 0.05). The transformation frequency declined with an extended pre-culture time, with a 6-day pre-culture resulting in a decline of the transformation frequency to 51.11%. Thus, a 2-day pre-culture was used to test the effects of the following parameters on the transformation frequency.

### Effects of *A. rhizogenes *cell density on transformation frequency

**Figure 6:**
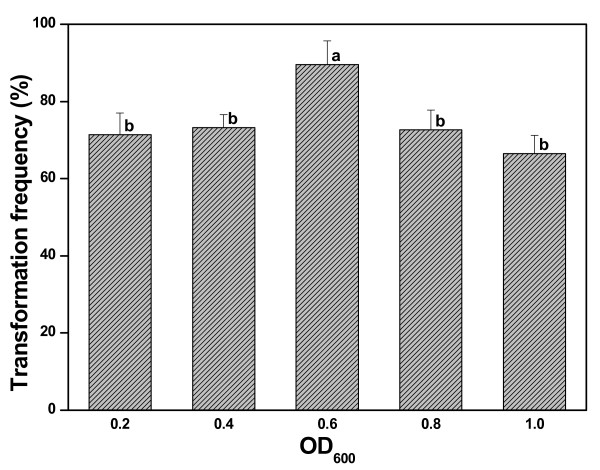
**Effect of *A. rhizogenes *cell density on transformation frequency**. *A. rhizogenes *cell density prior to inoculation was measured at OD_600 _nm. Column bars with the same letter are not significantly different at P < 0.05 as determined using LSD test. The experiment was performed in independent triplicate and each experiment contained about 30 samples.

The growth status of *A. rhizogenes *may influence its virulence, and thereby the transformation frequency. To assess it, stem sections with one node, which were precultured for two days, were infected with different density of *A. rhizogenes *culture corresponding to OD_600 _= 0.2, 0.4, 0.6, 0.8 and 1.0, respectively. They were subsequently treated as described for the standard procedure in Methods. The highest transformation frequency (89.64%) was obtained when *A. rhizogenes *cultures at the late-log stage were used, corresponding to OD_600 _= 0.6. At this OD_600_, transformation frequency increased significantly (P < 0.05) over all other tested cell concentrations (Figure 6).

### Effects of co-cultivation conditions on transformation frequency

**Figure 7:**
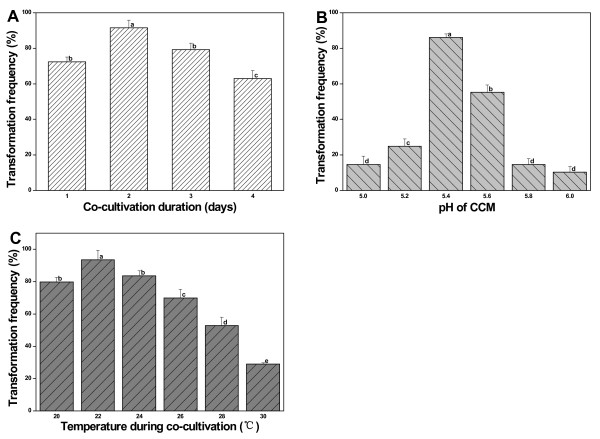
**Effects of co-cultivation conditions on transformation frequency**. Effects of duration of co-cultivation (A), pH of CCM (B) and temperature during co-cultivation (C) on transformation frequency were determined. Column bars with the same letter are not significantly different at P < 0.05 as determined using Fisher's LSD test. The experiment was performed in independent triplicate and each experiment contained about 30 samples.

After infection, the explants were placed on the CCM to allow T-DNA transfer from the plasmid into plant cells. Several parameters concerning the co-cultivation were tested in order to assess their impact on transformation frequency. For co-cultivation duration, the stem sections with one node were precultured for two days, infected with *A. rhizogenes *corresponding to OD_600 _around 0.6 and then placed on CCM (pH5.4) at 24°C for 1, 2, 3 or 4 days. After this, the explants were placed on 1/2 MS medium for hairy root production. As shown in Figure 7A, the highest transformation frequency (91.54%) was achieved with a 2-day co-cultivation. The transformation frequency was lower at both shorter and prolonged co-cultivation. To test the effect of the pH of the CCM, the standard procedure as mentioned in Methods was used except that the pH of CCM was tested at 5.0, 5.2, 5.4, 5.6, 5.8 and 6.0. A CCM pH level of 5.4 was found to be optimal, which led to the transformation frequency of 86.15%. CCM pH below or above 5.4 resulted in the decrease of transformation frequency, with the lowest being 10.31% at a pH of 6.0 (Figure 7B). Growth temperature affects the virulence functions of many pathogenic bacteria [22]. To determine the influence of temperature during co-cultivation on transformation frequency, the standard procedure was used except that temperatures of 20°C, 22°C, 24°C, 26°C, 28°C and 30°C during co-cultivation were tested. It was found that 22°C was the optimum temperature for co-cultivation, with transformation frequency being 93.59% (Figure 7C). The transformation frequency markedly decreased with an increase in temperature, dropping to 52.84% and 28.93% when the temperature was 28°C and 30°C, respectively.

### Hygromycin can be used as an efficient selection marker during plant regeneration

**Figure 8:**
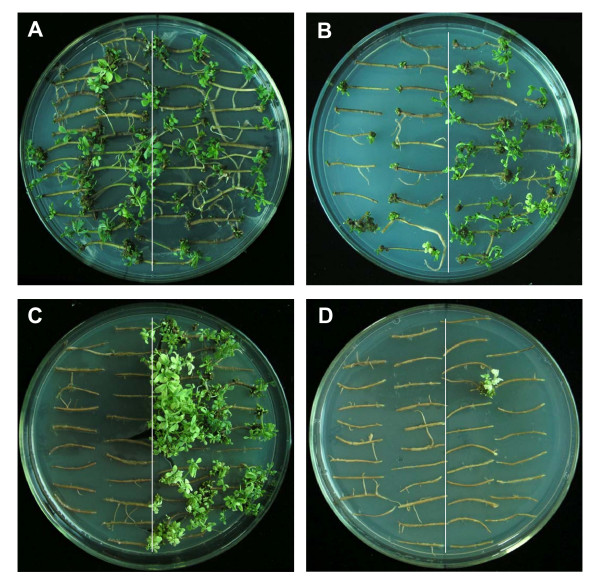
**Effects of hygromycin during plant regeneration**. Hygromycin was added into the RM in the final concentrations of 0 (A), 2 (B), 4 (C) and 6 mg/L (D), respectively. The plate was divided into two regions. About 20 positively transgenic root segments were put onto the right half and similar numbers of control roots were put onto the left half. The pictures were taken 4 weeks after inoculation. Hairy roots developed by *A. rhizogenes* harbouring no binary vector were used as negative control.

The effect of hygromycin on plant regeneration was also assessed. As shown in Figure 8, the regeneration frequency declined with an increase in hygromycin concentration. All roots can differentiate into shoot buds in RM without hygromycin and no difference was observed between transgenic and negative control roots (Figure 8A). When 2 mg/L hygromycin was added, 100% of the transgenic roots and still about 70% of the negative transgenic roots differentiated (Figure 8B). When 4 mg/L hygromycin was added into the RM, all negative control roots died. However, about 80% of the transgenic roots could still differentiate (Figure 8C). Few transgenic roots survived and differentiated into shoot buds when 6 mg/L hygromycin was added into the RM (Figure 8D). As all negative control roots died when 4 mg/L hygromycin was added and all regenerated plantlets were GUS positive, it can be concluded that 4 mg/L hygromycin is efficient to select transgenic plants during plant regeneration. In addition, this assay indicates that hygromycin can be directly used for selecting transgenic hairy roots without prior GFP or GUS detection.

### Validation of the gene function test system

**Figure 9:**
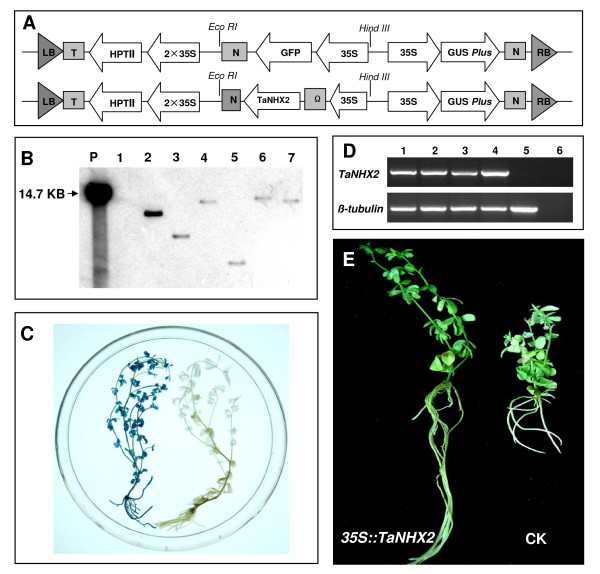
**Analysis of transgenic *TaNHX2 *events**. Schematic representation of the T-DNA regions of pGFPGUS*Plus *(upper panel) and pCMTaNHX2 (lower panel) (A). The relative location of GUS *Plus*, *HPT II *and *TaNHX2 *are shown. LB, left border; T, polyA site; 2×35S, double CaMV35S promoter; N, nopaline synthase (NOS) terminator region; RB, right border. Southern blot analysis of regenerated transgenic lines using a 728-bp *TaNHX2 *fragment as a probe (B). P, *Eco*R I-digested pCMTaNHX2 plasmid DNA; 1, negative control plant; 2–7, randomly selected transgenic regenerated plants. Transgenic *TaNHX2 *lines were identified by GUS staining in regenerated transgenic *TaNHX2 *plant (left) and a negative control (right) (C). *TaNHX2 *expression was analyzed by RT-PCR in *L. corniculatus *cv. *Superroot *transgenic lines (D). A specific PCR product of 728 bp (upper panel) was detected in four randomly selected *TaNHX2 *(1–4) transgenic lines. 5, negative control; 6, PCR on a mixture of 1–5 RNA samples without reverse transcription. A 252 bp *beta-tubulin *fragment was amplified as an internal control (lower panel). Phenotypes of representative *TaNHX2 *transgenic (35S::*TaNHX2*) and control (CK) *L. corniculatus *plants after treatment with 150 mM NaCl for 15 days (E). All negative control plants were regenerated from hairy roots developed by *A. rhizogenes *harbouring no binary vector.

**Figure 10:**
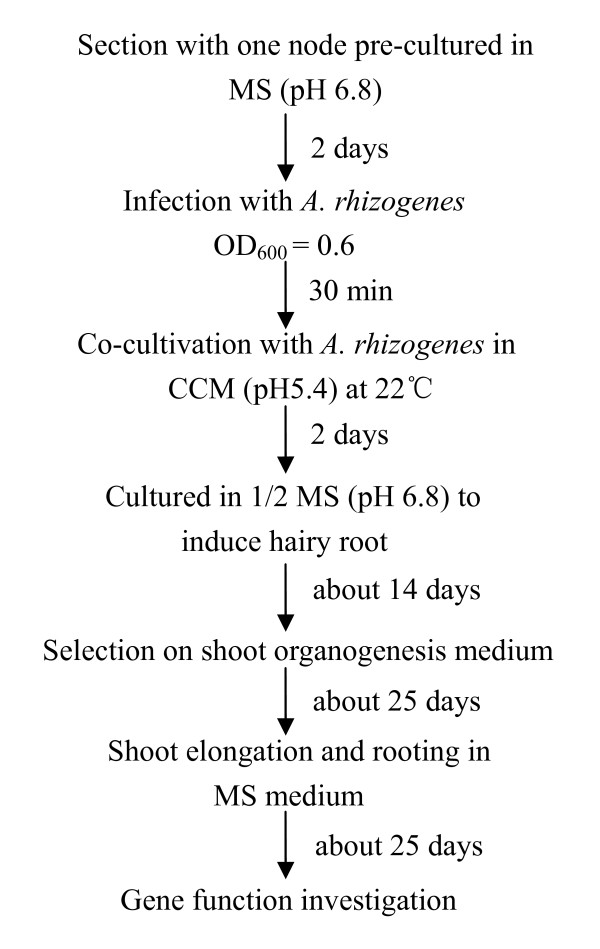
**A flowchart for *A. rhizogenes*-mediated transformation of *L. corniculatus *cv. *Superroot***.

In order to validate the gene function investigation system developed in the present study, pCMTaNHX2 was constructed (Figure 9A, lower panel). Transgenic hairy roots were obtained using stems section with one node as explants, 2-day pre-culture, infection with *A. rhizogenes *at OD_600 _= 0.6, co-cultivation on CCM (pH 5.4) at 22°C for 2 days. When all these optimal parameters were used, the transformation frequency could achieve 92%. Transgenic *L. corniculatus *plants were obtained in two and a half months (Figure 10). Southern blot analysis was performed to identify the transgenic events. Genomic DNA of regenerated plants was digested with *Eco*R I which cuts only once within the T-DNA. Restriction-digested DNA was then blotted and hybridized with a 728 bp DIG-labelled *TaNHX2 *fragment as a probe. As shown in Figure 9B, the six randomly selected transgenic regenerated plants showed a single integration event of the *TaNHX2 *gene thereby confirming their transgenic nature. No hybridization signal was observed in the control plant. GUS staining of the regenerated plantlets, with an example shown in Figure 9C, confirmed that T-DNA of the binary vector was integrated into the plant genome and GUS was expressed. No GUS expression was observed in the control plant. Four independent transgenic lines were randomly selected and the expression levels of *TaNHX2 *were monitored by reverse transcription-PCR (RT-PCR). *Beta-tubulin *(AY633708) was used as the reference gene. As expected, the transgenic *TaNHX2 *lines 1–4 expressed *TaNHX2*, whereas no expression was detected in the control plants (Figure 9D). In order to rapidly obtain a large number of transgenic *TaNHX2 *plants for salt tolerance assays, the plantlets regenerated from individual hairy root were cut into stem segments with one or two nodes and then inserted into MS medium for rooting. After 10–13 days, 90% segments produced roots. To test the salt tolerance of transgenic *Superroot *plants overexpressing *TaNHX2*, ten plantlets of each independent transgenic *L. corniculatus *line and the negative control were used. An example is shown in Figure 9E, the control plants grown for 15 days on MS medium (pH 5.8) containing 150 mM NaCl bleached, roots were stunted and plants were arrested in their growth. In contrast, the transgenic *Superroot *plants over-expressing *TaNHX2 *survived and exhibited healthy growth.

## Discussion

The production of transgenic plants is useful for investigating gene functions [23]. The rapid ongoing progress in functional genomic studies has increased the demand of highly efficient transformation systems for legumes [24]. The development of an efficient genetic transformation technology will facilitate physiological and molecular biology studies in *L. corniculatus *and the transgenic *Superroot *system will also be useful as a plant expression factory [14].

High-frequency production of transgenic plants relies on the highly efficient T-DNA delivery from *Agrobacterium *into plant cells [24], selection of transgenic cells and plant regeneration [25]. In the present study, *A. rhizogenes *K599 [5,8,9] harbouring pGFPGUS*Plus *[26] was used to optimize the transformation of *Superroot*-derived *L. corniculatus *plants. pGFPGUS*Plus *is an efficient transformation vector with two reporter genes, *GFP *and *GUS*, simplifying the identification of the transfer events. Hygromycin, an efficient selection agent for plant transformation [25], has been proved to be efficient in selecting the positive transgenic plants during plant regeneration in the present study too. As a matter of fact, only transgenic roots were able to differentiate into shoots and most of the transgenic roots produced plantlets when 4 mg/L hygromycin was added into the RM as shown in Figure 8. As all the plantlets able to regenerate on this selection medium expressed GUS and GFP, we propose that hygromycin can be used to select positively transgenic plants directly without GFP or GUS detection. This direct hygromycin selection saves time and reduces contamination.

The simplicity and high efficiency of the *Superroot *regeneration system [14] and the highly efficient selection system using pGFPGUS*Plus *make T-DNA delivery from *Agrobacterium *into plant cells a pivotal step in transgenic *Superroot *plant production. T-DNA delivery from *Agrobacterium *into plant cells is a complicated process which is influenced by many parameters such as *Agrobacterium *strain [11,27], pre-culture duration [15,21], explant type [15,16], temperature [10,22] and co-cultivation duration [15,17]. Evidently, not all bacteria are virulent to given host plant cells and not all plant cells are competent for infection and regeneration [28]. Thus, the improvement of bacterium virulence and plant cell competence would enhance T-DNA delivery into plant cells. In the present study, the stem section with one node was identified as the most suitable type of explant as it allowed the highest transformation frequency compared with root, internode and leaf. This suggests that the susceptibility of explants to *Agrobacterium *is dependent on the physiological state of different tissues in the same plant. The highest transformation frequency was observed when stem section with one node was pre-cultured for 2 days prior to infection with *A. rhizogenes *and it declined with an extended pre-culture duration. A possible reason for this may be that long-time pre-culture decreased the viability of explants. Two days was also confirmed as the optimum co-cultivation duration whereas a 3 or 4-day co-cultivation may cause the overgrowth of *A. rhizogenes *leading to damage of the plant cells and consequently resulting in a low transformation frequency. On the other hand, a shorter co-cultivation time may disrupt *A. rhizogenes *cell proliferation, thereby reducing its virulence and leading to a low transformation frequency. These results are consistent with the reports in some other legumes, such as *Lathyrus sativus *[17], *Cicer arietinum *[29,30] and *Vigna mungo *[31]. Gene transfer to plant cell is a temperature-sensitive process [22]. The highest transformation frequency was found when the co-cultivation was carried out at 22°C in this study. High temperature (over 26°C) led to less efficient transformation. The defect in transfer at high temperatures may be due to a reduced functionality of the T-DNA transfer machinery [32] or due to the fact that high temperature leads to a reduced level of virulence protein and hence bacterial virulence [22].

The high-throughput production of transgenic plants in a short time is important for gene function investigation, especially for plants where plant regeneration is a 'bottleneck'. *Superroot*, which was selected from 11 960 seeds at a 65% germination rate of *L. corniculatus*, showed faster growth and more vigorous embryogenic plant production on hormone-free medium [12]. The easy and efficient regeneration system of *Superroot *makes it a useful tool in gene function studies. However, direct stable transformation of *Superroot *was unsuccessful, hence limiting its use. Recently, transgenic *Superroot *of *L. corniculatus *were regenerated from *Superroot*-derived leaves using *A. tumefaciens*-mediated transformation [14]. However, the system in question takes six months from gene transfer to PCR analysis and the transformation efficiency was low. To date, the time-frame for the production of transgenic plants remains to be shortened in most species of the legume family capable of being transformed. For example, in *L. japonicus*, production of transgenic plants from hairy root cultures requires about 5–6 months. Even for the improved *A. tumefaciens*-mediated hypocotyl transformation, 4 months are needed for plant regeneration [33]. For *Medicago truncatula*, it generally takes 4 months to get transgenic plants [11]. In contrast, the obtainment of transgenic *Superroot *plants through the *A. rhizogenes*-mediated transformation described here requires only about two and a half months. Furthermore, as every transgenic root originates from a single cell [34,35] and represents an independent transformation event, a great numbers of transformants can be obtained and analyzed in a relatively short period of time [9]. For *Superroot *in *L. corniculatus*, many plantlets can be obtained from one transgenic hairy root on the selective RM. Moreover, it is easy for *L. corniculatus *to propagate in culture, starting from shoot tips and node sections [36]. Roots from regenerated transgenic plants can also easily differentiate into shoots. Thus, this system is convenient for getting large numbers of transgenic *L. corniculatus *plants in a short time. All the following characteristics allow *A. rhizogenes*-mediated transformation of *Superroot*-derived *L. corniculatus *plants to be considered as a useful platform for gene function investigation in *L. corniculatus*: 1) highly efficient and abundant production of transgenic hairy roots when *Superroot*-derived *L. corniculatus *plants are infected with *A. rhizogenes *K599; 2) regeneration of transgenic hairy roots into plantlets in one month; 3) fast and simple propagation process for *L. corniculatus*.

To validate this platform for gene function investigation, transgenic *Superroot*-derived *L. corniculatus *plants overexpressing *TaNHX2 *were obtained via the optimized *A. rhizogenes-*mediated transformation system developed in the present study and their salt tolerance was assessed. Transgenic *L. corniculatus *survived and grew in MS medium supplemented with 150 mM NaCl whereas the control plantlets bleached and exhibited an inhibited growth. With the method described here, it took about three months to assess the function of *TaNHX2 *on the whole plant level and thus may save a lot of time in comparison to similar studies in other plant species [11,33].

*A. rhizogenes*-mediated hairy root production has been widely used for root biology. Coupled with the fast, simple and highly efficient regeneration system of *Superroot*-derived *L. corniculatus *plants, we developed a rapid and highly efficient platform for gene function investigation in *L. corniculatus *and validated its utility by assessing the function of *TaNHX2*. However, some shortcomings of this system still remain. *L. corniculatus *is allopolyploid, self-sterile and a poor seed producer [13] and may therefore be of limited use for studies of genes involved in processes such as flower development. Nevertheless, this system should still be recommended for many other research fields, such as stress physiology, secondary metabolic biology and root biology.

## Conclusion

A rapid and highly efficient production of transgenic *Superroot*-derived *L. corniculatus *plants through *A. rhizogenes*-mediated transformation was developed, combining the simplicity and efficiency of the *Superroot *regeneration system and the highly efficient *A. rhizogenes *mediated transformation. The transformation frequency can reach 92% based on the detection of GUS activity. This system was improved by validating some parameters which may influence transformation frequency. The fast and highly efficient transformation and regeneration system of *Superroot*-derived *L. corniculatus *provides a powerful tool for gene function testing in *L. corniculatus *in a short time.

## Methods

### Plant material

*Superroot *culture of *L. corniculatus *was provided by Dr. Ryo Akashi and was propagated in liquid MS medium. Roots were then placed onto MS medium with 0.5 mg/L 6-benzylaminopurine (6-BA) for shoot induction. The shoots were excised and transferred to solid hormone free MS medium for seedling growth and rooting [12,13].

### Bacterial strain and binary vector

Cucumopine-type *A. rhizogenes *strain K599 with pGFPGUS*Plus *was used to transform *Superroot*-derived *L. corniculatus *plants. This binary vector has two reporter genes: β-glucuronidase (uidA) and *GFP*, both of which are controlled by the constitutive cauliflower mosaic virus (CaMV) 35S promoter. The *GUS *reporter gene contains a catalase intron inside the coding sequence to ensure that expression of glucuronidase activity is derived from eukaryotic cells. The hygromycin phosphotransferase (HPTII) gene was also located between T-DNA borders, allowing the hygromycin selection of positive transformants. *A. rhizogenes *K599 which lacks the binary vector was used as negative control for the transformation.

### Hairy root induction

*A. rhizogenes *K599 with pGFPGUS*Plus *was grown in dark at 28°C on agar-solidified LB medium supplemented with 50 mg/L kanamycin. To get fresh cells, a single bacterial cell colony was inoculated in liquid LB medium (20 ml) containing 50 mg/L kanamycin, cultured overnight and diluted with 1:1000 liquid LB medium. Segments of *L. corniculatus *cv. *Superroot *plants were immerged into the *A. rhizogenes *culture and shook (50 rpm) at 25°C for 30 min. The explants were dried on sterile filter paper and then transferred to CCM which was covered with a sterile filter paper. The CCM consisted of 1/10 MS, 3.9 g/L morpholino ethanesulfonic acid, 150 mg/L cysteine and 150 mg/L dithiothreitol. After co-cultivation, the explants were washed three times with sterile water and immerged in liquid 1/2 MS medium containing 250 mg/L cephalexin and 250 mg/L carbenicillin. The blot-dried explants were transferred to solid 1/2 MS medium with 250 mg/L cephalexin and 250 mg/L carbenicillin for hairy root induction.

### Plant regeneration

Two-week old hairy roots, which were produced at the wounding sites of explants after they were transferred to solid 1/2 MS medium, were cut off and placed onto RM for shoot induction. The shoots were transferred to hormone free solid MS medium for shoot elongation and rooting.

### Detection of *GUS *and *GFP *in transgenic plants by PCR

Genomic DNA was extracted from regenerated plants and *GUS *and *GFP *fragments were amplified by PCR to detect the transgenic events. The following sets of primer pairs were used for amplification. *GUS*: 5'-GATGATAGTTACAGAACCGACG-3' (forward), 5'-CATTCGGAATCTCCACGTTAC-3' (reverse).*GFP*: 5'-GTAAACGGCCACAAGTTCAGCG-3' (forward), 5'-TCGTCCATGCCGAGAGTGATCC-3' (reverse). 5 ng plasmid DNA was used as template for the amplification of positive control. Genomic DNA of regenerated plants derived from hairy roots induced by *A. rhizogenes *K599 harbouring no binary vector was used as negative control. The PCR conditions were as follows: 10 min at 94°C and 35 cycles of 30 s at 94°C, 1 min at 60°C for *GUS *or 62°C for *GFP *and 45 s at 72°C, followed by a final extension at 72°C for 10 min. The products of expected size for the two genes were verified by 2% agarose electrophoresis and SYBR Green staining.

### Southern blot analysis

Genomic DNA was extracted from regenerated plants. Approximately 40 μg of DNA from each sample was digested overnight with *Hind *III. 200 ng *Hind *III digested p35SGFPGUS*Plus *plasmid DNA was used as positive control. DNA fragments were separated by electrophoresis in 0.8% agarose gel, transferred to a Hybond-N^+ ^(Amersham Biosciences) nylon membrane, and cross-linked by baking at 80°C for 2 h. The DIG-dUTP (Roche) labelled *GUS *gene (750 bp) was used as a probe and hybridization was carried out according to the manufacture's instruction of DIG high prime DNA labeling and detection starter kit II (Roche). A regenerated plant from hairy root developed by *A. rhizogenes *harboring no binary vector was used as negative control.

### Histochemical localization of GUS and GFP expression

GUS staining was performed as previously described [37]. Briefly, the hairy roots and young seedlings were cut off and transferred to GUS staining solution (50 mM sodium phosphate at pH 7.0, 10 mM EDTA, 0.1% Triton X-100, 1 mg/ml X-Gluc, 0.1 mM potassium ferricyanide, 0.1 mM potassium ferrocyanide and 20% methanol) and incubated at 37°C for 0.5–12 h. The staining solution was then removed and the tissues were destained by washing several times in 70% ethanol. GUS detection was observed on an Olympus BX51 microscope. GFP expression of the hairy roots and regenerated plants was detected by confocal laser scanning microscope (Leica, TCS SP2). A regenerated plant from hairy root developed by *A. rhizogenes *harboring no binary vector was used as negative control.

### Protein extraction and Western blot for GFP detection

Plant tissue was homogenized in liquid N_2 _and the powder was transferred into a 1.5 ml Eppendorf tube containing 100 μl extraction buffer of 50 mM Tris-HCl (pH8.0), 120 mM NaCl, 10% glycerol, 0.2% Triton X-100, 5 mM phenylmethanesulfonyl fluoride, 10 mM dithiothreitol and 0.1% sodium dodecyl sulfate (SDS) and the tube was shaken vigorously at 4°C. After vortexing, the sample was centrifuged at 13,000 rpm for 30 min and the supernatant was used for the Western blot assay. The samples were boiled for 5 min in sample buffer and then centrifuged at 10,000 g for 10 min. The supernatant was separated by 12% SDS-polyacrylamide gels. Proteins were transferred to a pyroxylin membrane (Amersham Biosciences) and GFP was detected using polyclonal anti-GFP antiserum (Sigma). A regenerated plant from hairy root developed by *A. rhizogenes *harboring no binary vector was used as negative control.

### Determination of hygromycin selection level in plant regeneration

Roots of transgenic plants identified by GFP and GUS detection were cut into 2 cm fragments and placed onto RM containing different concentrations of hygromycin (0, 2, 4 and 6 mg/L) to find the optimum hygromycin for selection. Roots of transgenic plants derived from hairy roots that were induced by *A. rhizogenes *K599 harbouring no binary vector were used as negative control.

### Evaluation of parameters influencing transformation efficiency

A range of parameters were evaluated including explant type (root, internode, leaf, stem section with one node), pre-culture duration in MS medium (0, 1, 2, 3, 4, 5 and 6 days), OD_600 _value of the *A. rhizogenes *cell culture (0.2, 0.4, 0.6, 0.8 and 1.0), duration of co-cultivation (1, 2, 3 and 4 days), pH of CCM (5.0, 5.2, 5.4, 5.6, 5.8 and 6.0), temperature during co-cultivation (20, 22, 24, 26, 28 and 30°C). Referring to previous reports on *A. rhizogenes*-mediated transformation [10,11,38] and also *A. tumefaciens*-mediated transformation [11,15,39], the standard procedure, which was used to optimize parameters influencing the transformation frequency in the present study, was as follows. Stem sections with one node were precultured for two days (except for explant type parameter assay, in which four types of explant as mentioned above were used as explants and precultured for one day). They were then infected with an *A. rhizogenes *cell culture grown to an OD_600 _of 0.6 and co-cultivated on CCM (pH5.4) at 24°C for two days. After co-cultivation, the explants were washed with sterile water to remove excess *Agrobacterium*, and placed on 1/2 MS medium for hairy roots production. This standard procedure was used in all optimization assays, the only variable being the parameter to be optimized. The related description of each parameter assay is presented in the corresponding part of the Results section. All the parameters were tested in three independent transformation experiments and each experiment included three replicates, which consisted of about 30 explants each.

### Validation of the gene function testing system by assessing the function of *TaNHX2 *in transgenic Superroot-derived *L. corniculatus *plants

The *TaNHX2 *cassette was double-digested by *Eco*R I and *Hin*d III from pBIN438-*TaNHX2*, and then subcloned into the *Eco*R I/*Hin*d III site of pGFPGUS*Plus*. The resulting binary construct was named pCMTaNHX2 and introduced into *A. rhizogenes *strain K599 via electroporation. Transgenic *TaNHX2 L. corniculatus *plants were obtained using the method developed in the present study. Genomic DNA of randomly selected transgenic plants and a control plant was digested by *Eco*R I and used in Southern blot analysis in order to confirm the transgenic event using a DIG-labelled *TaNHX2 *fragment (728 bp) as a probe. 200 ng *EcoR *I digested pCMTaNHX2 plasmid DNA was used as positive control. GUS staining was also carried out to identify the transgenic events and the transformation frequency was calculated by the percentage of GUS positive hairy roots. The transgenic hairy roots were placed on the RM for shoot buds induction. The expression levels of *TaNHX2 *were detected by RT-PCR using *L. corniculatus beta-tubulin *as an internal control. Briefly, total RNA was extracted using Trizol (Invitrogen) according to the manufacturer's instruction from four randlomly selected *TaNHX2 *transgenic lines and four control plants. cDNA synthesis was carried out using the RNA-free DNase-treated RNA following the instruction of the SuperScript III First-Strand Synthesis System (Invitrogen). 2 μl first strand cDNA was used as template for PCR amplification. For negative control, a mixture of equal amount of cDNA from four control plants was used. The following sets of primer pairs were used for amplification: 5'-ACACTATTTGGTGCCGTTGG-3' (forward) and 5'-CTTCCAACCAGAACCAACCC-3' (reverse) for *TaNHX2*; 5'-TCTGATACTGTTGTGGAGCCT-3' (forward) and 5' TGGGATCAGATTCACTGCTAG-3' (reverse) for *beta-tubulin*. For *TaNHX2*, 35 cycles were used and for beta-tubulin, 28 cycles were used. In order to assess the salt tolerance of transgenic *Superroot L. corniculatus *overexpressing *TaNHX2*, individual positive transgenic *TaNHX2 *plants identified by GUS staining and also RT-PCR detection were cut into stem segments with one or two nodes and then inserted into MS medium for rooting. After 10–13 days, 90% segments produced roots. A regenerated plant from hairy root developed by *A. rhizogenes *harboring no binary vector was used as negative control. Ten plantlets of each independent transgenic *L. corniculatus *line and the negative control were transferred to MS medium (pH5.8) containing 150 mM NaCl. About 15 days later, the distinguishing phenotypes between the transgenic *TaNHX2 *plants and negative control plants were assessed.

### Data processing

The transformation efficiency was calculated by the percentage of GUS positive explants over total explants having hairy roots. All data were presented as mean ± standard deviation (SD). The effects of parameters on the transformation efficiency were validated by Fisher's LSD test at P < 0.05 level. All the experiments were conducted in independent triplicate and each experiment contained about 30 samples.

## List of abbreviations

RM: regeneration medium; CCM: co-cultivation medium; DIG: digoxigenin; LSD: least significant difference; RT-PCR: reverse transcription-PCR; 6-BA: 6-benzylaminopurine; CaMV: cauliflower mosaic virus; HPTII: hygromycin phosphotransferase; SDS: sodium dodecyl sulphate; SD: standard deviation.

## Authors' contributions

BJ performed all the experimental procedures, data analysis, drafted and revised the manuscript. WH provided technical and partial financial support and revised the manuscript. CW took part in tissue culture. BL participated in GFP assay, GUS analysis, Western blot, figures preparation and manuscript revising. WL constructed pCMTaNHX2 and performed partial salt tolerance assay. SS assisted in plant material providing. YB revised the manuscript. TH supervised the study, revised the manuscript critically and gave financial support to the study. All authors read and approved the final manuscript.
